# Providing technical assistance to ministries of health: lessons learned over 30 years

**DOI:** 10.9745/GHSP-D-13-00121

**Published:** 2013-11-14

**Authors:** Steven Solter, Catherine Solter

**Affiliations:** a Management Sciences for Health, Cambridge, MA, USA; b Pathfinder International, Watertown, MA, USA

## Abstract

Pursuing true country ownership for effective programs requires a long-term approach involving persistence, patience, keen understanding of counterparts' perspective, deference, building trust, focus on priorities, technical competence, and sustained optimism.

## INTRODUCTION

Beginning in 1976, we have each spent more than 30 years providing technical assistance (TA) to ministries of health (MOH), chiefly at the central level but also at the provincial level. More than 20 of those years were spent providing long-term assistance—that is, a year or more of continuous residence as a ministry advisor—in Afghanistan, Cambodia, Indonesia, and the Philippines. We have also provided short-term assistance to health ministries in many other countries. We both have worked for nonprofit organizations that provide technical assistance in public health to developing countries.

Over this period we have encountered an enormous variety of situations in the health ministries where we worked—major differences in cultural values, political pressures, priorities, and personalities among our ministry counterparts—and yet we feel that there was some consistency across the various countries in terms of what worked and what did not and why. We have learned a great deal from making almost every imaginable mistake at one time or another. This brief paper is an attempt to distill some of the major lessons we have learned (with examples) in order to contribute to discussion among those trying to provide the best technical assistance they can.

We have identified 10 lessons that we believe are among the most relevant and important. Some may seem self-evident; others are less so. The evidence must, by the very nature of the work, be largely subjective and anecdotal. A randomized field trial of technical assistance is not feasible. What we describe is based on our own experiences, which are not necessarily generalizable. However, in our discussions with other advisors over the years, we have found our experiences to be quite typical. Therefore, we propose these 10 principles to assist providers of technical assistance to ministries of health in helping governments make progress in health care.

## THE TOP 10 LESSONS LEARNED

(In no specific order, but with particular relevance to long-term TA)

1. **When you begin work as a technical advisor, your 2 immediate, overriding objectives are to establish trust and to demonstrate that you can add value**. Most of our ministry counterparts have been extremely polite, hospitable, and respectful, and they were not critical of our actions (or inactions) even when we deserved to be criticized. But that didn't mean they trusted us. We had to earn their trust, which takes time. An important caveat regarding establishment of trust is the importance of knowing where your loyalties lie and being up front about them. In most cases your loyalty is divided among ministry counterparts, the donor, the organization that employs you, your colleagues working with you as part of a technical assistance team, and the people of the country you are trying to serve. It can be very confusing and difficult when, for example, the donor expects you to represent them to the ministry at the same time that the ministry expects you to represent them to the donor. You are caught in the middle, trying to be honest and fair to both sides. There is no simple answer (other than to be honest and straightforward with all parties). There is one thing, however, that you should avoid if at all possible, and that is to have a hidden agenda. For example, we remember a donor that had a large quantity of contraceptives that were about to expire and wanted us to convince our counterparts to accept them as a donation. This made it difficult to do our job—to provide the best possible advice to the ministry.
  Proving that your presence adds value can be a challenge, especially when your ministry counterparts are well aware of your high salary (compared with theirs) and are often unhappy about the fact that donor money is going to you instead of to them. It is imperative that you understand your counterparts' agenda and the constraints that they are facing so that what you offer is perceived as truly helping them to achieve their objectives. Very often senior health officials are facing political pressure from higher-ups, and they may ask for your help with something that isn't in your job description. A typical example is when you are asked to draft a presentation they need to make (in English) at an international conference, and they need it tomorrow! If you do it, without complaint and on time, you can go a long way towards convincing them that you actually do add value.
  It is imperative that you understand your counterparts' agenda and the constraints that they are facing.
2. **It's their country, not yours, and they make the decisions, not you.** This should be obvious, but it's surprising how often ministry counterparts think of you as a “foreign expert” and therefore more knowledgeable than they are. Fortunately, the trend is now going the other way, as more and more health ministry staff members obtain master's and doctoral degrees and no longer always feel that they know less than you do.
  An important responsibility of being a technical advisor is to assemble evidence-based options for decision-makers to review and decide which option is best under the circumstances. As an advisor you can present your point of view, but the decision isn't yours to make. A case in point was when the head of the maternal and child health unit of a MOH asked one of us to help with planning the country's first National Immunization Day. Our advice was to include only oral polio and vitamin A for the first round. We thought including too many antigens would be complicated and could result in delays as mothers wait in line in the heat with crying babies to register for DPT (diphtheria, pertussis, and tetanus), BCG (bacille Calmette-Guérin), and measles vaccines. Furthermore, adding these antigens requires needles and syringes and trained staff, whereas oral polio and vitamin A capsules can be given by villagers with a brief training beforehand. Our advice had logic on its side, but it turned out to be dead wrong. The ministry and the villagers organized things so well that all the antigens were given at the same time with nary a hitch. It turned out to be a good thing that we *didn't* make the decision.
3. **Understand the full context of your counterparts' situation, and base your assistance and advice on an understanding of where he or she is coming from.** For example, it is often months or even years before advisors fully understand how health ministries handle procurement. In a province where we worked, it took 46 signatures to procure an item vital for public health work, making the process incredibly time-consuming. But we didn't realize why everything moved so slowly, and so much of our advice was irrelevant.
  Another example, from a different country, involved concerns of health officials over potential accusations that they were selling government items in the market and pocketing the money. In order to protect themselves from such charges, the safest thing to do was to lock up everything and not allow anything to get out of sight. As a result, midwifery kits were locked up and never used, and empty cooking oil tins by the thousands were kept in a warehouse for years as proof that the oil had actually been distributed rather than sold in the bazaar.
  Similarly, we once saw a guard burying a broken chair in the ground. When we asked him why he was doing that, he said that the chair was broken beyond repair, but, if he threw it out, in the future someone might accuse him of selling it. If that accusation were ever made, he could go to the spot where the chair was buried, dig it up, and prove that he was innocent.
  A final example involves a ministry whose procurement regulations were so restrictive that purchasing the pens and notebooks for a training workshop had to go out for bids. Since we didn't realize that this was happening, we weren't able to suggest alternative (and much faster) ways of obtaining the pens and notebooks. As an advisor, if you're not fully aware of such constraints at all levels of the system, your advice is likely to be impractical or ignored.
4. **Out of sight, out of mind—it's important to “be there.”** There are perils in being a long-term advisor, but there are also risks for those providing short-term technical assistance at a ministry of health. One problem faced by the long-term advisor is that after several years he or she becomes “part of the furniture” and is no longer listened to as carefully or taken as seriously as in the first 1 or 2 years. Most foreign advisors have a limited repertoire of messages and concerns that they feel passionate about. When those messages and those passions have been expressed over and over, our counterparts tend to tune us out. But if you stay the course and stick around for a number of years, it's possible to make a real difference in terms of policy and other initiatives that can have real impact. The first rule of public health technical assistance is “be there.”
  One problem faced by the long-term advisor is that after several years he or she becomes “part of the furniture.”
  Those providing short-term technical assistance face an even tougher challenge: Once they're out the door and on the way home, they tend to be forgotten and their advice ignored (until the next time they come). One distinguished short-term consultant, a former medical school dean and noted researcher, came as a World Health Organization (WHO) consultant to advise on cancer control. He was told that he was the seventh such consultant over the past decade and that none of the recommendations of his 6 predecessors had ever been implemented. He was absolutely determined to change that pattern, and he did everything humanly possible to ensure that his cancer control recommendations would be carried out. Alas, it was not to be. His recommendations were ignored like all the others. But then, 8 years after he left, the ministry became actively engaged in cancer control, dug up the old recommendations, and actually implemented some of them. The lesson here was that the ministry decided when it was ready to take on a new initiative, and no consultant was going to get them to do it before they were ready. Short-term advisors need to be aware of this risk, but we don't know of any way to guarantee that their recommendations will be followed. You can try to identify a ministry official who will champion your suggestions, but beyond that it is difficult to overcome the “out of sight, out of mind” syndrome.
5. **WHO does a great job in setting international standards and guidelines, but sometimes ministries of health defer to international guidelines rather than adapt those guidelines to fit the special circumstances prevalent in their country.** Over the years both of us have been struck by the “WHO paradox.” WHO does a superb job of developing international standards, guidelines, and protocols. Most ministries of health in developing countries follow those standards very closely, and the guidance has made an enormous difference in quality of care and impact on mortality. The problem is that, even though WHO always insists that each country must adapt the standards and guidelines to fit their own particular circumstances, in reality many countries hesitate to make any changes out of fear that they'll be violating international norms.
  One example occurred when a Geneva-based WHO expert came to a ministry meeting to discuss the use of misoprostol in home births. The ministry had already conducted a field trial in which 1,000 pregnant women received misoprostol tablets from the local volunteer female community health worker (CHW) without a single instance of the drug being given at the wrong time or diverted for use as an abortifacient. Despite this strong evidence of safety and despite the fact that the maternal mortality ratio was extremely high in the country, the WHO advisor strongly advised the ministry not to allow CHWs to give families misoprostol prior to delivery on the grounds that the CHWs didn't have enough training to do it safely (although their training lasted 6 months). The ministry backed down and sharply reduced its plans to scale up misoprostol distribution. Many other examples could be cited to make the same point. The challenge for ministries and for us as advisors is to listen to WHO's advice and take it seriously, but also to recognize that a country's unique situation often calls for a modification in the policies and guidelines recommended by WHO.
6. **Country “ownership” rarely happens, but it has to be a top priority.** Although donors and international agencies continually tout the concept of “country ownership,” we have rarely seen it in practice. The main problem, in our experience, is that ministries of health are often so dependent on donors for the bulk of their funding that they hesitate to oppose any suggestion by a donor, even when ministry officials feel it is not in the country's best interest. But donors often are focused on international priorities rather than the priorities of a particular country.
  Yet this is another situation where we think the trend is moving in a positive direction. In recent years health ministries have begun to assert themselves and be more vocal vis-à-vis donors to express their views about what may or may not be appropriate. We applaud this trend, but we are concerned that many ministries still lack basic skills in “donor management” and end up doing what the donors want rather than what they want.
  We also recognize, however, that doing what the donor wants is not always a bad thing, since in our view the donors are often right about priorities. For example, donors often advocate for CHWs to provide injectable contraceptives as well as community case management for diarrhea, pneumonia, and malaria. The ministry may resist these interventions on the grounds that only better trained health workers should be diagnosing and treating or giving injections. Regardless of who's right and who's wrong, ministry of health officials, not the donors, should “own” the policy decisions and interventions that follow. Unless the ministry makes the decision, it won't “own” its implementation or its effects.
7. **Keep it simple and focused.** We have seen over and over again the mistakes we've made when we (and our fellow advisors as well) propose options and scenarios more complicated than they need to be, and when we and our counterparts try to do too many things at once. It is demonstrably true that we can do 20 things badly or 3 things well. Very often, for political reasons, our ministry colleagues have difficulty prioritizing, since there is a political constituency for everything they want to do. Everything is vitally important, and nothing can be left out. Possibly the most important thing we can do as advisors is to help our ministry colleagues focus on a few top-priority activities and interventions and help them place lower-priority activities on the back burner. We can sometimes facilitate this process because we are “outside” the political, ethnic, and cultural system they inhabit, and, if anyone criticizes them, they can blame it on the advisor, who doesn't know any better.
  Possibly the most important thing we can do as advisors is to help our ministry colleagues focus on a few top-priority activities and interventions.
  Keeping it simple is necessary because a simple concept can obtain political traction among higher-level decision-makers, whereas overly complicated proposals usually get nowhere. Also, nearly everyone values keeping things simple, and it is a way that we, as advisors, can “add value” in our day-to-day work. Sometimes our counterparts think that a more complicated proposal or paper is better because it is more sophisticated and looks like journal articles they have seen over the years. It is our job to try and disabuse them of that notion.
  Keeping it simple is necessary because a simple concept can obtain political traction among higher-level decision-makers.
8. **When you see evidence of corruption, act on it.** All long-term advisors and many short-term consultants encounter evidence of corruption at one time or another among their ministry colleagues. A common response is to ignore it, since one is rarely certain that it has actually occurred, and, in any case, if you raise the issue, you are likely to get in hot water and possibly lose your job, or even be asked to leave the country. But to do nothing perpetuates the problem. The question is: what to do? As a foreigner, you have to tread very carefully, but there is usually an existing mechanism that can be used to report what you have found.
  One of our experiences illustrates the problem. The project involved paying community-based volunteers a small amount of money to identify suspected tuberculosis (TB) cases and having their sputum checked at a health facility with a trained microscopist (in the early 1980s). The volunteer was paid for each sputum-positive individual and then paid a second time (more money than the first payment) when the patient converted after treatment to sputum-negative. The program started out as a rollicking success, with hundreds of new sputum-positives identified, treated, and cured. But the money to pay the villagers came from the donor to the central MOH, which passed it on to the provincial ministry, which in turn gave it to the ministry at the district level and finally to the sub-district, where the villagers were to be paid. Alas, by the time the funds were supposed to arrive at the sub-district, there was nothing left. Apparently, each level had taken its cut. Thus, all the money had been siphoned off, and there was nothing left to pay the villagers. Needless to say, the activity ground to a sudden halt. We didn't press the matter, fearing that doing so would jeopardize the project's other activities and also could poison relations with counterparts. But the end result was a failed activity and hundreds of people with active TB going undiagnosed and untreated. We should have pressed the issue.
9. **Help your counterparts collect the right information to provide evidence to their ministries; support “data for decision-making” at the local level.** We have found that “indicator creep” is a common disease—far more indicators than necessary are designated as crucial, and the number keeps growing. We remember an advisor working with MOH counterparts trying to reduce the number of indicators routinely reported from health centers throughout the country (this was around 1980, in the early days of computerization). Because the MOH was not experienced in inputting the data, every month the backlog grew larger, until there were enormous stacks of monthly reports not yet processed. But every time the advisor met with program managers (epidemiology, maternal and child health, tuberculosis, etc.), the list of indicators actually got longer, not shorter. The program managers kept discovering new indicators that they wanted to include in the monthly reports.
  “Indicator creep” is a common disease.
  Of course, indicator creep places an unnecessary burden on those collecting routine data at the local level. Sometimes we ourselves have been guilty of collecting too much data—a syndrome known as “Data Rich, Information Poor” or DRIP. The quantity of data can be overwhelming, and prioritizing becomes more difficult. But the crucial point, we've found, is this: most health workers at the local level see routine data collection as a burden and that what they are required to do is simply to report the data to the next highest level. They do not see the opportunity to use the data to figure out how to do a better job and improve their performance. We have seen many health centers shut their doors to patients at noon so that they can spend the afternoon collecting, recording, and reporting data. The data are then passed up the chain and eventually get included in obligatory reports that no one reads. The lesson we've learned is the importance of working with the health staff members at community and facility levels who actually collect the data to help them learn how they can use it themselves instead of only sending it to the next level. It is more important to use routinely collected data locally than to report it and forget about it.
10. **Manage expectations, but remain optimistic about what can be achieved within a typical 5-year project life cycle.** It frequently happens that donor-funded projects have unrealistic goals and objectives and that the expectations of donors and counterparts alike become impossible to satisfy. Part of the advisor's job is to help counterparts and donors to be realistic, especially about what can be achieved in a fixed and limited period of time, and what can truly become financially sustainable. Once again, the question of priorities becomes paramount; it is critical from the outset of the project to reach consensus among all stakeholders regarding what is of overriding importance and what isn't. Reaching this consensus early on can avoid a lot of grief at a later date. In one country where we worked, the project was very successful primarily because the ministry, the donor, and the major international organizations (especially WHO, the World Bank, and the European Union) all reached consensus early on and didn't waver in their commitments, despite the replacement of the original decision-makers with new ministers and new top-level staff in the partner agencies. Sadly, we have found that this level of agreement, partnership, and unwavering commitment is the exception rather than the rule.
  Despite all the setbacks that inevitably occur with any project, it is imperative that advisors remain upbeat; the glass should always be half-full rather than half-empty. One advisor we knew, who had more than 30 years of international health experience, was an absolute pessimist; he had seen hundreds of well-intentioned projects fail over the years, and he could provide multiple reasons why every project being considered would also fail. We're certain he was right about some of those proposed projects, but there needs to be a certain level of optimism, or otherwise nothing new or innovative will ever be tried. Fortunately, the MOH didn't always follow his advice and was able to implement a number of successful initiatives that he had advised against.
  The glass should always be half-full rather than half-empty.

## CONCLUSION

All these “principles” and lessons learned are based on the experience of expatriate advisors with ministries of health. As MOH advisors are now increasingly more likely to be citizens of the countries they are advising, some of these principles will need to be revised or eliminated. But we believe many of them hold true, regardless of the nationality of the advisor. Although our experiences have been exclusively in developing countries, we also feel that many of the principles and lessons apply to many developed countries, as well.

We hope that these principles and the associated anecdotes will help those engaged in technical assistance in the health sector, so that their assistance is as effective as it can be and achieves the greatest possible impact. Some of these principles may be relevant to project design as donors try to maximize their return on investment.

The hardest task of all for advisors and implementing partners alike is to make donor-funded projects financially sustainable. In 30 years of work as ministry advisors, we have not found any easy answer for that challenge. But we are optimistic about the future, as a new generation takes the helm in ministries of health around the world.

The hardest task of all is to make donor-funded projects financially sustainable.

